# Sugar-Coated Killer: Serotype 3 Pneumococcal Disease

**DOI:** 10.3389/fcimb.2020.613287

**Published:** 2020-12-23

**Authors:** Jennifer N. Luck, Hervé Tettelin, Carlos J. Orihuela

**Affiliations:** ^1^ Department of Microbiology, The University of Alabama at Birmingham, Birmingham, AL, United States; ^2^ Department of Microbiology and Immunology, Institute for Genome Sciences, University of Maryland School of Medicine, Baltimore, MD, United States

**Keywords:** *Streptococcus pneumoniae*, invasive pneumococcal disease, serotype 3, synthase-dependent pathway, vaccine escape, capsule production, wzy-dependent pathway

## Abstract

Capsular polysaccharide (CPS), which surrounds the bacteria, is one of the most significant and multifaceted contributors to *Streptococcus pneumoniae* virulence. Capsule prevents entrapment in mucus during colonization, traps water to protect against desiccation, can serve as an energy reserve, and protects the bacterium against complement-mediated opsonization and immune cell phagocytosis. To date, 100 biochemically and serologically distinct capsule types have been identified for *S. pneumoniae*; 20 to 30 of which have well-defined propensity to cause opportunistic human infection. Among these, serotype 3 is perhaps the most problematic as serotype 3 infections are characterized as having severe clinical manifestations including empyema, bacteremia, cardiotoxicity, and meningitis; consequently, with a fatality rate of 30%–47%. Moreover, serotype 3 resists antibody-mediated clearance despite its inclusion in the current 13-valent conjugate vaccine formulation. This review covers the role of capsule in pneumococcal pathogenesis and the importance of serotype 3 on human disease. We discuss how serotype 3 capsule synthesis and presentation on the bacterial surface is distinct from other serotypes, the biochemical and physiological properties of this capsule type that facilitate its ability to cause disease, and why existing vaccines are unable to confer protection. We conclude with discussion of the clonal properties of serotype 3 and how these have changed since introduction of the 13-valent vaccine in 2000.

## Introduction

*Streptococcus pneumoniae* (*Spn*), also known as the pneumococcus, is a Gram-positive encapsulated bacterium commonly identified by its lancet-shaped diplococcal morphology. An opportunistic inhabitant of the nasopharynx, humans are the only natural host for *Spn*. Problems arise in colonized individuals when pneumococci ascend the Eustachian tubes to the middle ear where they can cause otitis media. Alternatively, when pneumococci are aspirated into the lower respiratory tract and induce pneumonia. Notably, the likelihood of either of these events is starkly increased among very young children, those who are immunocompromised, the elderly, and those who are experiencing or have recently experienced viral infection (Harboe et al., 2009; Klugman et al., 2009; Infante et al., 2015). Critically, and once established in the lower respiratory tract, the pneumococcus can cause bacteremia, i.e. invasive disease. This occurs in ~30% of hospitalized individuals (Center for Disease Control, 2015). As result *Spn* is a leading cause of bacterial sepsis following pneumonia (Mayr et al., 2014). Bloodborne *S. pneumoniae* are not restricted to the vasculature and able to invade other organs including the central nervous system to cause meningitis and myocardium to cause cardiac complications (Brown et al., 2014; Shenoy et al., 2018; Africano et al., 2020). Not well appreciated, but a critical aspect of pneumococcal disease, is that survivors of severe infection, most often the elderly, often experience considerable sequelae. These include onset of frailty, cognitive declines, loss of independence, increased risk for adverse-cardiac events, and reduced lifespan (Lucas et al., 2016; Brooks and Mias, 2018). In the United States, it is estimated there were 31,400 (9.6 cases per 100,000 individuals) cases of invasive pneumococcal disease in 2018 with 3,480 directly attributable deaths (Centers for Disease Control and Prevention, 2018). Worldwide the number of serious pneumococcal infections is thought to exceed 1–1.5 million per year (World Health Organization, 2007). Thus, this bacterium, which has garnered the alias of “The Old Man’s Friend”, is a serious cause of morbidity and mortality worldwide and a leading health problem.

## Role of Capsule

Capsule is a gelatinous external layer produced by bacteria to protect against phagocytosis and other external hazards. In addition to being a common feature of almost all clinical isolates of *Spn*, capsule is a common feature of most extracellular bacterial pathogens (Nelson et al., 2007; Standish and Morona, 2015; Zhensong Wen, 2015). Bacterial capsules are polymers, with most versions being composed of a polysaccharide. Yet, capsule can be made of other molecules such as in *Bacillus anthracis* where it is a polypeptide (Zhensong Wen, 2015). Polysaccharide capsules are oligomers synthesized in sequential manner by a group of enzymes that link monosaccharides and other moieties (e.g., acetyl groups) together, and then assemble them as a repeating chain. In most *Spn*, capsule strands are attached to the bacterial surface via covalent bonds to peptidoglycan. One exception to this is serotype 3 pneumococcal capsule, which instead uses non-covalent interactions with phosphatidylglycerol (Yother, 2011; Cartee et al., 2005; Standish and Morona, 2015; Larson and Yother, 2017). Along such lines and given the extensive genomic variability in the enzymes responsible for construction of the oligosaccharide unit, the biochemical variability that exists between bacterial capsule types including within *Spn* is tremendous. As of 2020, 100 biochemically and serologically distinct versions of *S. pneumoniae* capsule have been identified each of which is encoded by distinct sets of enzymes arranged together in the capsule operon (Ganaie et al., 2020). Importantly, some serotypes, such as serotype 3, have historically been associated with a much higher attack rate and/or morbidity than other serotypes (Harboe et al., 2009; Grabenstein and Musey, 2014). Along such lines, observations by Sandgren et al. as well Brueggemann et al. suggest that the biochemical properties of the capsule directly contribute to virulence, as isogenic capsule switch mutants with the same accessory genome content were shown to have differences in virulence relative to the capsule type that was produced (Brueggemann et al., 2003; Sandgren et al., 2004).

Unencapsulated pneumococci are capable of nasopharyngeal colonization and due to selective pressure from the current vaccines (see below) are thought to be an emerging population. Unencapsulated pneumococci are capable of causing localized infections such as sinusitis, conjunctivitis and keratitis, as well as otitis media (Reed et al., 2005). Importantly, in some instances, unencapsulated isolates have had the capsule cassette replaced with either *aliC*, *aliD*, or *pspK* (Park et al., 2012; Geno et al., 2015). AliC and AliD are oligopeptide binding proteins and their deletion has pleiotropic effects on the bacterium including modulation of surface adhesins (Bradshaw et al., 2018). PspK is a homologue of the pneumococcal adhesin Choline binding protein A (Keller et al., 2013). In other instances where *Spn* is unencapsulated, there are mutations in the capsule operon that preclude capsule synthesis (Park et al., 2012; Geno et al., 2015). As is detailed below the absence of capsule profoundly changes the interactions the bacteria have with its host (Magee and Yother, 2001).

During nasopharyngeal colonization, individual pneumococci must avoid entrapment in mucus (Magee and Yother, 2001; Nelson et al., 2007). It is now recognized that capsule, specifically its electronegativity, acts to electrostatically repel mucus, which is also negatively charged, and thereby avoid entrapment and subsequent expulsion. Nelson et al. showed that isogenic strains carrying capsule with a net negative charge avoided mucous entrapment better than versions carrying neutral-charged capsule or an unencapsulated control (Nelson et al., 2007). Similarly, Li et al. showed that capsule electronegativity influenced the serotype’s nasopharyngeal carriage prevalence (Li et al., 2013). A requirement for capsule becomes starkly apparent during pneumonia and invasive disease (Avery and Dubos, 1931). In a mouse model of intraperitoneal challenge and sepsis, the 50% lethal dose of an unencapsulated derivative of a serotype 3 isolate was 5.0 × 10^7^ colony forming units (CFU), as opposed to the 50% lethal dose of its parent wildtype strain of 1 CFU (Watson and Musher, 1990). The reason for this is that unencapsulated pneumococci are exquisitely susceptible to opsonization and phagocytosis by host factors and immune cells, respectively (Oss, 1978; Kim et al., 1999; Wartha et al., 2007). C-reactive protein, components of the alternative complement cascade, ficolin, surfactant, and pre-existing antibodies against conserved host proteins generated as result of past colonization events or infection, individually and in complementary fashion opsonize pneumococci for phagocytosis (Kim et al., 1999; Kraiczy and Wurzner, 2006; Tian et al., 2009; Hyams et al., 2010a; Kjaer et al., 2013). Capsule is inhibitory of phagocytosis as it modulates recognition by the alternative complement pathway (Hyams et al., 2010a; Hyams et al., 2010b). Additionally, capsule prevents the receptors on immune cells from binding to these molecules even though they are bound to the bacterial surface (e.g., Fc receptor with cell wall bound antibody) (Hyams et al., 2010a; Shenoy and Orihuela, 2016). Thus, the generation of antibody against the capsule itself, is critical for clearance of this pathogen during disease, though the amount of antibody required for clearance varies dependent on the serotype (Alonso De Velasco et al., 1995; Choi et al., 2016).

From the bacterium’s perspective, encasement within capsule is not always optimal, as capsule also inhibits interactions between bacterial adhesins and host epithelial cells, a step required for colonization and disease (Moscoso et al., 2006; Bootsma et al., 2007; Sanchez et al., 2011a; Qin et al., 2013). One solution that the bacteria has is phase-variation where the bacterium stochastically and at low frequency switches back and forth between a transparent (low capsule) and opaque (high capsule) phase (Kim and Weiser, 1998; Kim et al., 1999). The transparent version is selected for in the upper airway during colonization, where cell attachment is necessary. Whereas the opaque phase is favored in the lower respiratory tract and bloodstream, during which risk for opsonophagocytosis is greater (Kim and Weiser, 1998; Nelson et al., 2007). It is also now known that pneumococci can shed their capsule and quickly modulate their binding affinity. Using scanning electron microscopy, Hammerschmidt et al. showed that the binding of individual pneumococci to epithelial cells results in the loss of capsule from its surface (Hammerschmidt et al., 2005). One explanation for this are findings by Kietzman et al. which showed that peptidoglycan-bound capsule is released by a hydrolase in response to the bacterium’s exposure to cationic antimicrobial peptides (Kietzman et al., 2016). In this instance, capsule shedding serves dual purpose, protecting the bacterium from these positive-charged membrane-damaging products and exposing otherwise capsule-covered bacterial adhesins which in turn allow for intimate interactions with host cells. This concept is in agreement with past findings by our group which showed not only does capsule influence the relative exposure of pneumococcal virulence determinants, but this also occurs in a capsule type dependent manner (Sanchez et al., 2011b). As such, surface proteins must be compatible with the biochemical properties of capsule to be effective. Evidence supporting this includes work by Kelly et al. which showed conversion of a strain from one serotype to the next does not consistently increase or decrease virulence, and instead this was genome dependent (Kelly et al., 1994; Mizrachi Nebenzahl et al., 2004; Bootsma et al., 2007). Thus, the interplay between capsule and other factors on the surface are crucial.

Whereas capsular polysaccharide (CPS) from the K1 serotype of *Porphyromonas gingivalis*, *Neisseria meningitidis*, and numerous other bacterial pathogens have been demonstrated to be pro-inflammatory (d’Empaire et al., 2006; Graveline et al., 2007; Zughaier, 2011), work by Tuomanen et al. found that purified CPS from *Spn* is not inflammatory on its own (Tuomanen et al., 1985). Pneumococcal capsule’s poor immunogenicity stood in stark contrast to the robust immune response elicited by purified pneumococcal cell wall, which is known to contain Toll-like receptor (TLR)-1/2 and TLR-2/6 binding tri- and di-acetylated teichoic and lipoteichoic acids, respectively. In fact, *Spn* capsule has been demonstrated to have a general anti-inflammatory role as it hinders the interaction of bacterial components, such as cell wall, with pathogen-associated molecular pattern (PAMP) receptors, such as TLRs. Evidence for this includes work by Kung et al. which showed that encapsulated pneumococci elicited less CXCL8 IL-8, a potent chemokine, from epithelial cells than isogenic unencapsulated mutants (Kung et al., 2014). These differences in capsule immunogenicity between bacterial species are likely the result of each capsule type’s distinct biochemical properties and in turn their ability to be recognized by the host (d’Empaire et al., 2006; Graveline et al., 2007; Zughaier, 2011; Kung et al., 2014).

Because capsule is highly abundant and surface exposed, antibody against capsule is typically highly opsonic and protective against invasive disease. Capsule is currently utilized as the principal antigen in multiple present-day vaccines against pathogens such as *Haemophilus influenzae* type B and *Salmonella enterica* serovar Typhi (Centers for Disease Control and Prevention, 2015). CPS is also the primary antigen in the three currently licensed vaccines against *Spn*. These vaccines include purified polysaccharides from the 10 (serotypes 1, 4, 5, 6B, 7F, 9V, 14, 18C, 19F, and 23F), 13 (plus serotypes 3, 6A, and 19A), and 23 (plus serotypes 2, 8, 9N, 10A, 11A, 12F, 15B, 17F, 20, 22F, and 33F) most virulent serotypes of *Spn*, respectively. The 10- and 13- valent versions composed of capsule conjugated to a protein carrier, the 23-valent being composed of purified CPS alone. Of note, the 10-valent conjugate vaccine is not approved for use in the USA but is extensively used in many other countries.

It is at this point important to note that CPS, not being a protein, is not presented by dendritic cells to CD4+ cells in context of MHC and is therefore a T-cell independent antigen. Key implications of this include that children under 5 fail to develop protective immunity against capsule following immunization (Bonten et al., 2015; Center for Disease Control, 2015). Moreover, immunization with purified polysaccharide elicits only a modest and short-lived, <5 years, protective response among adults (Butler et al., 1999; Center for Disease Control, 2015). It is for this reason that the 10- and 13-valent versions of the pneumococcal vaccine consist of CPS conjugated to a protein carrier. Processing of the protein/polysaccharide by antigen presenting cells now occurs in context of MHC-II presentation resulting in CD4+ T cell involvement, generation of long-lived memory B-cells, and robust immunogenicity in children (Malley et al., 2005; Center for Disease Control, 2015; Shenoy and Orihuela, 2016). The resultant antibody titers against these conjugated capsule types are not only typically sufficient to protect against invasive disease caused by these serotypes but also against nasopharyngeal colonization and thereby confer herd immunity by disrupting transmission (Andrews et al., 2014; Choi et al., 2016). Unfortunately, and as is discussed below, serotype 3 is an important exception as the amount of antibody required for protection is not elicited by the current conjugate vaccine (Choi et al., 2016). Other serotypes that require higher antibody concentrations for efficient opsonization include 1 and 5; albeit this is most likely the result of a combination of factors, not just the biochemical properties of capsule, such as the carriage of virulence determinants that block amplifying complement sensitivity (Hyams et al., 2013; Burton et al., 2017).

Finally, and in contrast to capsule-based vaccines, naturally acquired immunity against *Spn* is the result of antibody generated against the bacterium’s surface proteins rather than the capsule (Wilson et al., 2017). For this reason, and given prior exposures as children, healthy adults do not typically develop pneumococcal disease. Ongoing efforts to improve on the existing vaccines are focused on expanding the coverage of the 10- and 13-valent vaccine to include more serotypes, identification of conserved pneumococcal proteins that might be co-administered to provide protection against the serotypes not included in the vaccine, and even development of whole cell pneumococcal vaccines that would generate antibody and a T-cell response against conserved surface proteins (Xu et al., 2015; Pichichero et al., 2016; Pichichero, 2017; Campo et al., 2018; Businesswire, 2020; Feldman and Anderson, 2020).

### Unique Features of Serotype 3 and Its Importance in Disease

**Figure 1:**
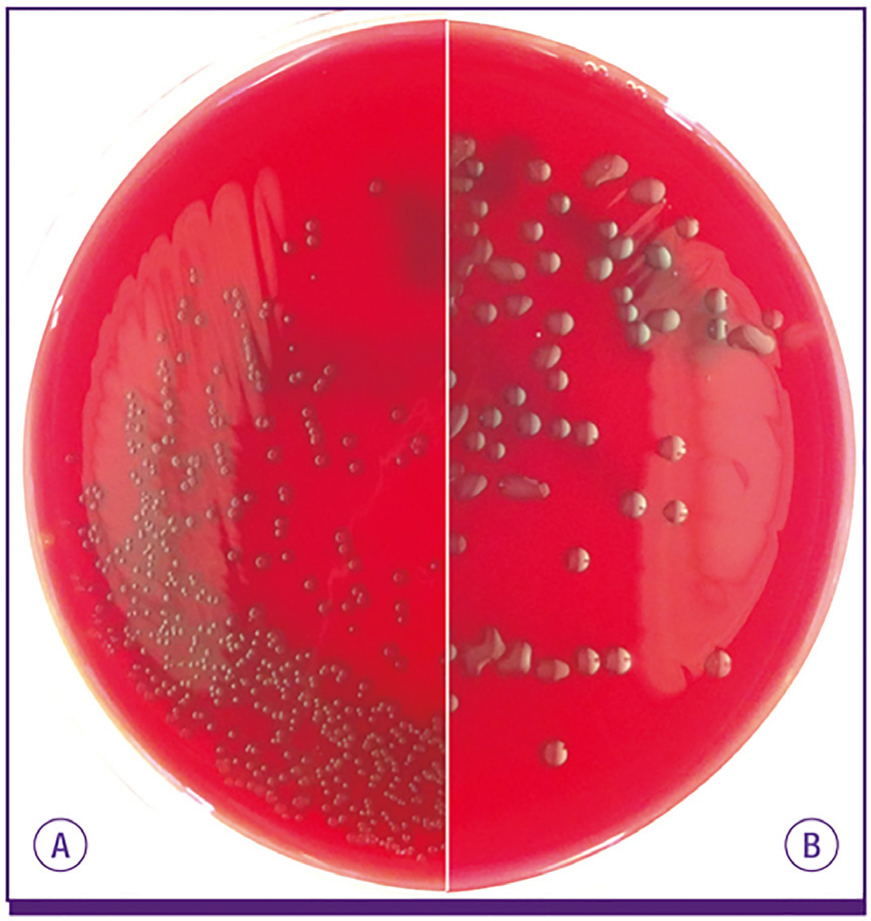
*S. pneumoniae* isolates expressing most capsule types make **(A)** small round colonies similar to doughnuts on blood agar plate, but **(B)** serotype 3 and 37 pneumococci develop characteristically large mucoid colonies. From Song et al. (2013).

Pneumococcal serotypes were named in numerical fashion based on the order they were identified. Thus, and in general, the lower number serotypes were initially more frequent causes of serious pneumococcal disease. This has changed as result of the conjugate vaccines which have reduced incidence of disease caused by the lower numbered serotypes, with now higher numbered serotypes becoming more prevalent as replacement strains. Clinical isolates producing serotype 3 capsule are distinct from most other pneumococcal isolates as they have a highly mucoid appearance and a wet phenotype when grown on plates (**Figure 1**). In contrast, other serotypes form more discrete colonies. Given their considerable difference in colony appearance, serotype 3 isolates were not initially recognized as being pneumococci (Howard and Perkins, 1901; Watson et al., 1993). In 1901, pathologists William Howard and Roger Perkins published a paper describing their isolation of an unfamiliar streptococcus from the abdomen of a young woman who had died as result of peritonitis (Howard and Perkins, 1901). The investigators observed the emblematic mucoid colonies, describing their appearance as “dewdrops” (Howard and Perkins, 1901). In their study, they recognized a series of others who had isolated bacteria with properties similar to their own and offered the name *Streptococcus mucosus*.

**Figure 2:**
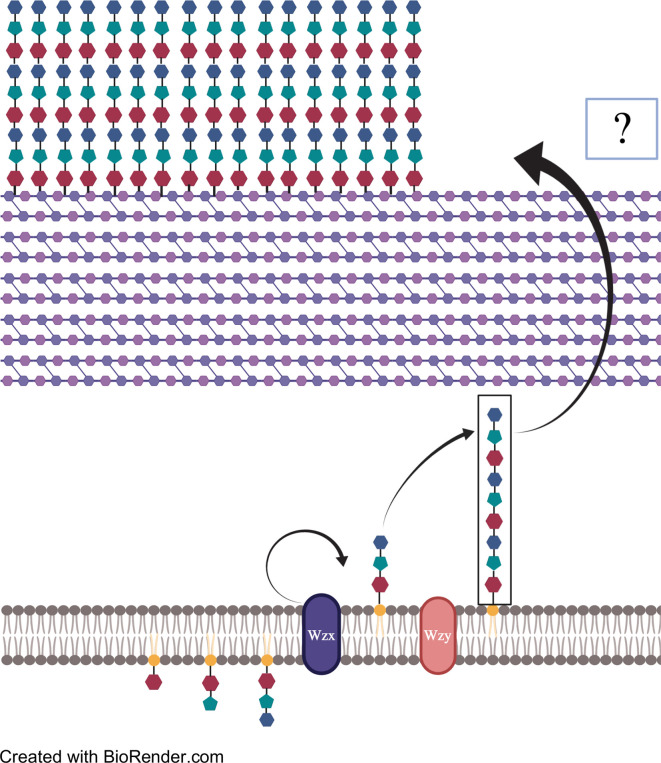
A pictorial explanation of Wzy-mediated CPS synthesis. Repeated patterns of monosaccharides are transported to and added to undecaprenylphosphate (Und-p). The polysaccharide strip is then flipped across the membrane by Wzx flippase and repeat segments are added onto the non-reducing end by Wzy polymerase. Once the desired length is reached, polysaccharide chains are exported from the cell membrane to the peptidoglycan by unknown means, where they bind covalently via glycosidic bond.

CPS production in pneumococci occurs by one of two mechanisms: Wzy-mediated or synthase-mediated synthesis. So far, Wzy-mediated capsule production has been observed in all but two pneumococcal serotypes; the two exceptions being serotypes 3 and 37 which instead rely on the synthase-mediated pathway (Yother, 2011; Standish and Morona, 2015; Zhensong Wen, 2015). The loci responsible for either mechanism is located at the same region within the *Spn* genome, between the *aliA* and *dexB* genes, except for serotype 37 which operates outside of the canonical CPS locus (Llull et al., 1999; Llull et al., 2001). Within the Wzy-mediated locus are the four highly conserved genes *cpsA*, *B*, *C*, and *D*. Genes encoding the serotype specific glucosyltransferases can be found next along with those encoding the conserved Wzy polymerase and flippase (Yother, 2011; Standish and Morona, 2015; Zhensong Wen, 2015). In Wzy-mediated synthesis, serotype-specific UDP-glycosyltransferases assemble a short polysaccharide chain with an enzyme-specific pattern inside the bacterial cytoplasm. The chain is then transferred to the Wzx flippase which reorients the chain into the periplasm (**Figure 2**). The short polysaccharide is then added on to the previously synthesized chain by the Wzy polymerase. Once the chain has reached a designated length, the polysaccharide is released and bound covalently to the cell wall (Yother, 2011; Standish and Morona, 2015; Zhensong Wen, 2015). Considerable diversity in the UDP-glycosyltransferases encoded within the capsule operon results in Wzy-mediated capsules having multitudes of monosaccharides ordered in specific patterns, the basis of serotypes (Standish and Morona, 2015; Geno et al., 2015). Serotype 3 CPS production utilizes synthase-mediated processes. Though the locus of serotype 3 is organizationally similar to that seen in strains that instead utilize Wzy-mediated synthesis, the CPS locus for synthase models contains multiple mutations and truncations that effectively silence many of the genes (Yother, 2011; Garcia et al., 1997; Standish and Morona, 2015). The only functional genes within a serotype 3 CPS locus are *cps3D*, which encodes UDP-Glc dehydrogenase; and *cps3S*, which encodes for the actual synthase.

**Figure 3:**
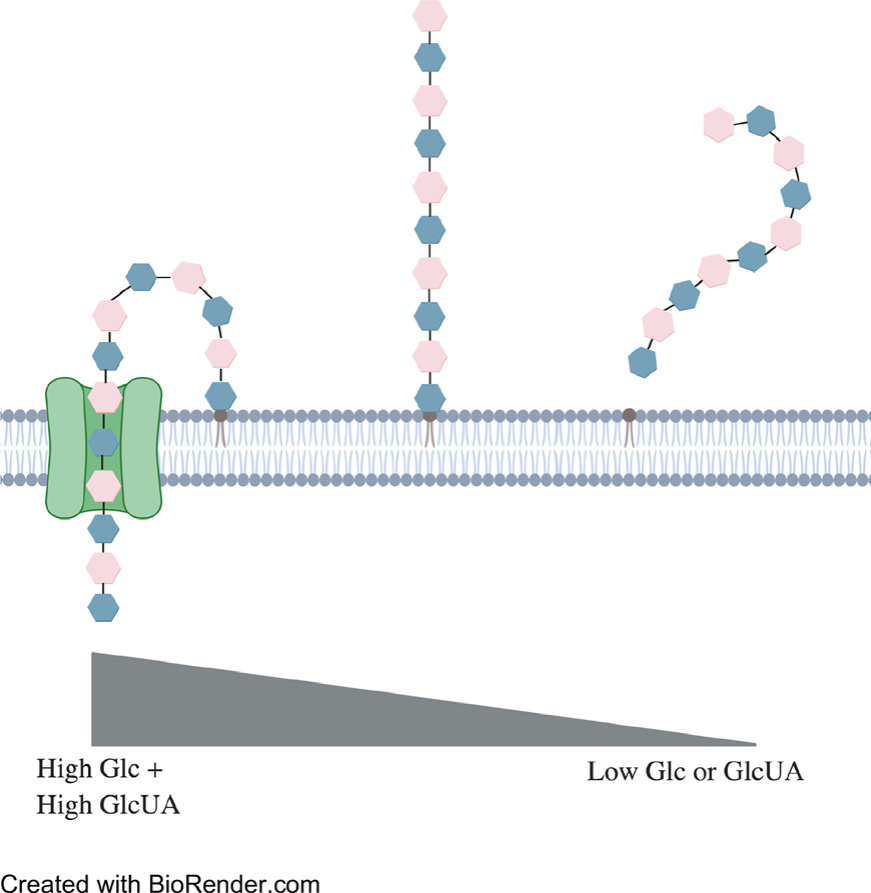
Representation of synthase-mediated CPS production. Synthesis begins when a glucose monosaccharide is transferred to an integral phosphatidylglycerol. Addition of a glucuronic acid is catalyzed by the synthase. This method repeats until concentrations of either sugar runs too low for synthesis to continue. The phosphatidylglycerol then dissociates from the synthase and disperses within the external cell membrane.

Synthase-mediated production of CPS is therefore much simpler than Wzy-mediated synthesis (Yother, 2011; Standish and Morona, 2015; Zhensong Wen, 2015). For example, serotype 3 contains only two monosaccharides, glucuronic acid and glucose, arranged in an alternating pattern, compared to the Wzy-mediated synthesis pathways which typically contain four to six monosaccharides per pattern (Standish and Morona, 2015). The sugars of synthase-mediated production are added directly onto the growing (non-reducing) end of the polysaccharide chain which is additionally exported and adhered to the external peptidoglycan via glycosidic bond (**Figure 3**) (Cartee et al., 2000; Forsee et al., 2006; Larson and Yother, 2017). CPS synthesis is continuous in serotype 3 strains and the polysaccharide chains only dissociate from the synthase when component concentrations are running low (Cartee et al., 2000; Ventura et al., 2006). Possible reasons for why capsule production is considerably greater for serotype 3 than other types include fewer steps in synthesis and therefore fewer checkpoints. Alternatively, this method of CPS production may not be as metabolically taxing on the bacterium.

### Serotype 3 Is a Major Cause of Human Disease

Even though serotype 3 is included in the both the 13- and 23-valent vaccines against *Spn*, it remains a major causes of serious human disease (Harboe et al., 2009; van Hoek et al., 2012; Grabenstein and Musey, 2014; World Health Organization, 2020). Notably, rates of disease attributable to serotype 3 have not declined since inclusion of serotype 3 in PCV13 (Centers for Disease Control and Prevention, 2015; Slotved et al., 2016; Katoh et al., 2017; Ladhani et al., 2018; Groves et al., 2019; Wijayasri et al., 2019; Goettler et al., 2020). Serotype 3 disease often has severe clinical manifestations: most commonly bacteremia-induced septic shock, meningitis, and pneumonia (Harboe et al., 2009; Silva-Costa et al., 2018). In some countries serotype 3 is also been linked to complicated pneumonia, or empyema, where the bacterium is found in the pleural cavity (Goettler et al., 2020). For all these reasons, serotype 3–influenced invasive disease boasts an ~30% mortality rate. This number climbs to 47% for individuals with multiple comorbidities (Charlson Index of +3), being most often the elderly (Harboe et al., 2009). Notably in a recent study by Africano et al., serotype 3 was independently associated with development of adverse cardiac events in hospitalized individuals (Africano et al., 2020). This was due to the bacterium’s propensity for bacteremia, an aspect of disease which confers the bacterium the opportunity to invade the myocardium (Ahl et al., 2013; Brown et al., 2014; Shenoy et al., 2018; Anderson et al., 2018). Thus, and despite exhibiting relatively low carriage rates among the population, serotype 3 associated opportunistic disease is consistently a major cause of IPD within young and old age groups with devastating consequences.

### Mechanisms of Vaccine Escape for Serotype 3

In a study by Choi et al., the amount of protection granted by the PCV-13 vaccine versus serotype 3 capsule production was investigated (Choi et al., 2016). Results from this experiment suggest that the profuse production and release of serotype 3 capsule overwhelms the protective capacity of antibody that is elicited by the vaccine. A measured 0.2 μl of serotype 3 culture supernatant containing the respective capsule was sufficient to abolish the antibody-mediated protection provided by the vaccine. In comparison, only 25 μl of supernatant from a serotype 4 isolate was required to reach that same point of neutralization. From these results, it was estimated that approximately eight times more antibody was required to confer protection against serotype 3 invasive pneumococcal disease (Andrews et al., 2014; Choi et al., 2016). In addition to inhibiting interactions of the bacteria with phagocytes, serotype 3 is able to escape from capsular antibody due to the fact that its capsule is not covalently attached to the bacterial surface, allowing it to be released in copious amounts to the extracellular milieu (Cartee et al., 2005; Choi et al., 2016). Capsule antibody therefore instead binds to shed capsule and is neutralized in its capacity to opsonize the bacteria itself. Antibody bound to the capsule on the bacterium’s surface would also be eventually released.

Bacterial surface components with negative electric charge can repel the like-charged immune cells (Oss, 1978). This aids the bacterium in avoiding NETs and phagocytosis, as well as complement particles released by phagocytic cells (Oss, 1978; Nelson et al., 2007). Many physical qualities of bacteria have been linked to the determination of cellular surface charge; one being capsule type (Li et al., 2013). With 100 biochemically distinct pneumococcal serotypes in existence, the level of surface charge can vary considerably. Surface charge is typically determined by measuring a cell’s zeta potential. Neutral charges exist between −10 and +10 mV. While charges of −30 and +30 mV make up the lower and upper extremes, respectively (Clogston and Patri, 2011). The lower the zeta potential, the higher the surface electronegativity and vice versa. Bacteria with lower surface charges (lower zeta potentials) generally perform better at avoiding phagocytosis and complement deposition than their neutral counterparts (Oss, 1978; Wilson et al., 2001; Li et al., 2013). Among such lines, Li et al. related lower zeta potential to higher rates of carriage in the nasopharynx. In his study, serotype 3 stood as the capsule type with the lowest average zeta potential (Li et al., 2013).

### Limitations Imposed by Serotype 3

As indicated, capsule is inhibitory of the adhesin and host ligand interactions that promote bacterial attachment. Studies with a capsule deficient version of serotype 3 strain A66.1 found its unencapsulated derivative to be 10^5^-fold more adhesive to host cells (Hammerschmidt et al., 2005). Furthermore, wildtype A66.1 recovered from interaction with HEp-2 cells showcased a decreased capsule expression level similar to that seen in the capsule deficient variant. Both observations support the notion that capsule shedding is necessary for interaction with host epithelia. Capsule has been also shown to be inhibitory of bacterial biofilm formation (Kim and Weiser, 1998; Hammerschmidt et al., 2005; Sanchez et al., 2011a; Qin et al., 2013). Presumably, capsule inhibits initial attachment of bacteria to a surface or the epithelia, it likely also interferes with bacteria to bacteria interactions. Interestingly, *in vitro*, serotype 3 has been found to rely on the formation of unencapsulated small colony variants (SCVs) for the formation of biofilms (Allegrucci and Sauer, 2007; Allegrucci and Sauer, 2008). These unencapsulated mutants have alterations in the genes encoding the capsule operon as result of oxidative stress imposed by the bacteria itself (Allegrucci and Sauer, 2007). These SCVs bind to the abiotic surfaces, and in turn are bound by fully encapsulated *Spn*. While this has not been shown to occur *in vivo*, it demonstrates that type 3 capsule most likely imposes challenges that the bacteria must overcome. Importantly, capsule is one component, along with other polysaccharides, of both *in vitro* and *in vivo* biofilm extracellular matrices (Hall-Stoodley et al., 2008). Thus, and although it is initially inhibitory of biofilm formation, it has an important role in the process.

Capsule, of course, is not the only determining factor of bacterial virulence. Investigations into variations of pneumococcal surface proteins such as PspA and CbpA (PspC) also showcased different levels of virulence and surface exposure when compared using isogenic WU2 (serotype 3) and TIGR4 (serotype 4) strains, respectively (Ren et al., 2003; Georgieva et al., 2018). We have shown, with the pneumococcal serine-rich protein PsrP, that the surface proteins which contain adhesive domains must project these through the capsule in order to function (Shivshankar et al., 2009; Sanchez et al., 2010). Thus serotype 3, being thickly encapsulated, puts in place unique parameters for the surface proteins it relies on for host-pathogen interactions.

### Population Genomics of *S. pneumoniae* and Serotype 3 Isolates

More than 20,000 whole genome sequences from isolates of *S. pneumoniae* are currently available in databases. A significant number of genomes were recently contributed by the Global Pneumococcal Sequencing (GPS) project (https://www.pneumogen.net/gps/) (Gladstone et al., 2019). The analysis of 20,027 genomes by Gladstone et al. was performed in the context of issues with current conjugate vaccines, including the concern about the emergence of non-vaccine serotypes. They aimed to study *S. pneumoniae* serotype, antibiotic resistance, and invasiveness in association with the overall genetic background of the isolates. They partitioned lineages into Global Pneumococcal Sequence Clusters (GPSCs) that were then associated with the features of interest listed above, as well as clonal complex (CC) and of course serotype (Gladstone et al., 2019; Lo et al., 2019; Gladstone et al., 2020). A CC groups related bacterial strains through the identification of allele variations or sequence types (STs) within seven highly conserved housekeeping genes (*aroE*, *gki*, *gdh*, *xpt*, *spi*, *ddl*, and *recP*) (Enright et al., 2001). For associations with GPSCs, CCs were defined as STs with single locus variant differences within the GPS dataset.

Serotype 3 has been the focus of recent genome-based studies that used GPSCs as the foundation (at least in part) and focused on the epidemiology and evolution of serotype 3 isolates in the US (Azarian et al., 2018), as well their prevalence in *Spn* carriage and in PCV13 evasion in the UK (Groves et al., 2019; Sheppard et al., 2019). In particular, CC180, an *S. pneumoniae* clone also known as Netherlands^3^-31 or PMEN31, is the major complex of serotype 3, containing a majority of clinical isolates and laboratory strains that have been sequenced and analyzed to date (Azarian et al., 2018; Groves et al., 2019). CC180, which is included in GPS lineage GPSC12, can be divided into 3 clades: clade I, which contains subclades Iα and Iβ, and clade II (Azarian et al., 2018; Groves et al., 2019). When the prevalence of isolates from each clade was investigated over a set timeframe, incidences of Iβ remained relatively unchanged while a switch in preference was observed between clades Iα and II (Azarian et al., 2018; Groves et al., 2019). Clade Iα, the predominant group in terms of isolates showed a slight decrease in population shortly after PCV13 introduction. Meanwhile, the population of clade II expanded, overtaking Clade Iα. By 2014, clade II made up ~41% of CC180-related pneumococcal isolates in the US. Up from ~20% just 4 years earlier in 2010 (Azarian et al., 2018). Interestingly, despite the noticeable increase in Clade II isolates and decrease in Iα isolates, no correlation was found between the introduction of PCV13 and Clade II expansion (Azarian et al., 2018). This suggested other means for clade II selection. Further investigation into the variation of several protein antigens gave insight into this matter. Genes encoding key virulence determinants such as NanA, PspA, and CbpA showed high levels of variability between the three clades. Variations in each of these genes has been shown to impact pneumococcal virulence in a variety of experimental conditions (Camara et al., 1994; Enright et al., 2001; Ren et al., 2003; Shivshankar et al., 2009; Sanchez et al., 2010; Georgieva et al., 2018; Azarian et al., 2018; Gladstone et al., 2019; Lo et al., 2019; Sheppard et al., 2019; Gladstone et al., 2020). Several variations in genes responsible for increased drug resistance, such as Tet32, were also observed within clades Iβ and II, but not within Iα isolates (Azarian et al., 2018; Groves et al., 2019). Azarian et al. hypothesized that these and other untested loci gave clade II the necessary competitive advantage to overtake clade Iα, though this has yet to be tested.

### Potential Strategies to Protect Against Serotype 3

The inability of PCV13 to elicit protective antibody against serotype 3 capsule suggests that a new alternative approach is necessary. Along such lines, immunization with conserved pneumococcal proteins has shown to confer protection against invasive disease caused by serotype 3 (Roche et al., 2003). Importantly the selected protein antigens must be conserved across CC180’s three distinct clades, be antigenic, be constitutively expressed at high level *in vivo*, and be demonstrated to confer protective immunity (Ginsburg et al., 2012; Moffitt and Malley, 2016). Candidate proteins for this include PspA and pneumolysin among others (Xu et al., 2015; Pichichero et al., 2016). Ideally, the selected proteins should also confer protection against the serotypes not included in PCV13 or other expanded vaccine formulations, thereby conferring protection against all *Spn*. In similar fashion, a whole-cell based vaccine using killed *Spn* that expresses conserved proteins and elicits protective antibody would confer similar serotype-independent protection against *Spn* (Pichichero, 2017; Campo et al., 2018). In both instances, these new vaccines would expand protection against serotypes that, although individually infrequent, together inflict a significant burden of human disease (Butler et al., 1999; Ginsburg et al., 2012; Pichichero et al., 2016; Moffitt and Malley, 2016; Pichichero, 2017; Campo et al., 2018). It is noteworthy that efforts are ongoing to expand on the current conjugate vaccines. A 15-valent conjugate vaccine, now including serotypes 22F and 33F, is currently undergoing phase 3 clinical trials and appears to generate more antibody against serotype 3 than the 13-valent version (Businesswire, 2020). Whether this will be sufficient to reduce overall serotype 3 disease remains to be determined.

## Conclusion

**Figure 4:**
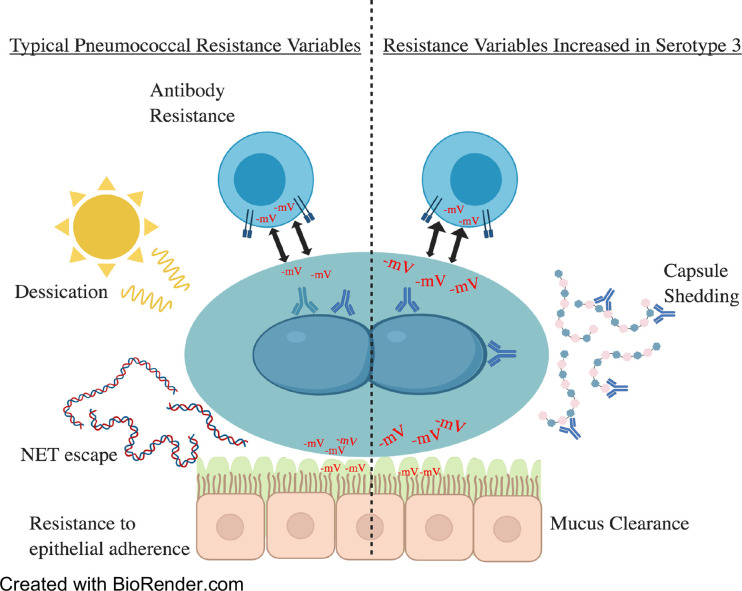
A summary of the topics covered within this review. To the left are virulence factors commonly seen and utilized by pneumococcal serotypes. On the right are virulence factors increased in serotype 3 strains. These include but are not limited to electronegativity and capsule production/shedding. Electronegativity has a variety of effects on virulence as demonstrated above. Higher electronegativity acts as a repellent of immune cells and mucin, allowing for increased escape of immune responses and nasopharyngeal colonization by mucin evasion. Increases in capsular polysaccharide shedding in serotype 3 have been hypothesized to overwhelm the immune responses by sequestering antibodies and minimizing the deposit of antibody on the cell surface.

Throughout this article, we have discussed the vital role of capsule on pneumococcal pathogenesis and how unique feature of serotype 3 provides the bacterium with enhanced virulence and an intrinsic ability to resist vaccine induced antibody (**Figure 4**). Whereas the biochemical properties of serotype 3 indeed influence its interactions with host cells, more simplistically, the copious amount of capsule that are produced by serotype 3 pneumococci overwhelms the 0.35 µg/ml antibody threshold provided by the current conjugate vaccine formulation (Andrews et al., 2014; Choi et al., 2016). Physiological properties of the capsule itself, such as surface electronegativity, and its ability to confer protection against host factors are also an explanation for why disease caused by serotype 3 has such severe disease manifestations (Nelson et al., 2007; Harboe et al., 2009; Li et al., 2013; Grabenstein and Musey, 2014). The bountiful production of serotype 3 capsule both benefits and challenges the bacterium, preventing agglutination by the nasal mucin and phagocytosis by host immune cells while simultaneously deterring adherence to the host epithelium (Allegrucci and Sauer, 2007; Allegrucci and Sauer, 2008). As such, obtaining a general understanding of the variability and basis for selection within serotype 3 regarding its complementary virulence determinants becomes an important goal (Azarian et al., 2018; Groves et al., 2019). A new approach seems to be necessary in order to prevent serotype 3 disease. Vaccines that generate antibody against conserved pneumococcal proteins have been tested in the past with reasonable efficacy and may hold the key to a stark decrease in pneumococcal-related incidents (Roche et al., 2003; Ginsburg et al., 2012; Moffitt and Malley, 2016). Moving forward serotype 3 is likely to continue being a major medical problem for some time until a new alternative prophylactic approach is approved.

## Author Contributions

JL wrote the first draft of the paper. CJO, JL, and HT contributed to the writing, editing, and direction of the paper. All authors contributed to the article and approved the submitted version.

## Funding

HT and CJO received grant support from NIH grants AI114800 and AI146149.

## Conflict of Interest

The authors declare that the research was conducted in the absence of any commercial or financial relationships that could be construed as a potential conflict of interest.
